# Clinical Examination of the Urine and Urinary Diagnosis

**Published:** 1901-12

**Authors:** 


**Clinical Examination of the Urine and Urinary Diagnosis.** By J. Bergen Ogden, M.D. Pp. 416. Philadelphia and London: W. B. Saunders and Company. 1900.

This is a well-written book, and gives a very complete account of its subject. One of its chief features is that it not only presents a full account of the chemical, microscopic, and spectroscopic investigation of the urine, which forms the first part of the book, but also in a second part describes the characters of the urine as altered in disease. Both parts are well done, the first being full in detail, and a trustworthy guide to the clinical examination of the urine; and the second, shorter than the first, giving a concise account of the changes in the urine in all forms of disease, and laying stress on points important in differential diagnosis. The nomenclature and division of diseases of the kidneys themselves is a little different from that commonly adopted in English text-books, and we should like to see some directions as to the order in which the various tests for proteid should be used clinically, in order to quickly obtain most information from them as to the variety of proteid present. The whole work is carefully written, is well abreast of the times, is well illustrated, clear in its directions and descriptions, and will be found a useful book of reference for the practitioner's bookshelf. It is nicely printed, and forms a handsome volume, reflecting credit on both author and publisher.

